# Author Correction: Applying the Jellium model to octacarbonyl metal complexes

**DOI:** 10.1038/s42004-020-0297-y

**Published:** 2020-04-08

**Authors:** Kun Wang, Chang Xu, Dan Li, Longjiu Cheng

**Affiliations:** 1 Department of Chemistry, Anhui University, 230601 Hefei, Anhui P.R. China; 2 Anhui Province Key Laboratory of Chemistry for Inorganic/Organic Hybrid Functionalized Materials, 230601 Hefei, Anhui P.R. China

**Keywords:** Chemical bonding, Chemical physics

Correction to: *Communications Chemistry* 10.1038/s42004-020-0285-2, published online 26 March 2020.

The original version of this Article incorrectly designated Kun Wang as a corresponding author and listed their e-mail address in error. This has now been corrected in the PDF and HTML versions of the Article.

