# Supplementary material for: Applying the Jellium model to octacarbonyl metal complexes
Source: Commun Chem. 2020 Mar 26;3:39. doi: 10.1038/s42004-020-0285-2 (PMC9814474; doi:10.1038/s42004-020-0285-2)
Supplement: Supplementary file 1 — Supplementary Information [file 42004_2020_285_MOESM1_ESM.pdf]

Supplementary Information of

Applying the Jellium model to octacarbonyl metal complexes $[M(CO)_8]^q$ (M, q = Ca, 2-; Sc, 1-; Ti, 0; V, 1+; Cr, 2+; Ba, 2-)

Kun Wang[1,2], Chang Xu[1] , Dan Li[1] and Longjiu Cheng[1,2]*

Supplementary Table 1. The calculated vibrational frequencies (cm^{-1}) of $[M(CO)_8]^q$ and $[M(CO)_7]^q$ (M, q = Ca, 2-; Sc, 1-; Ti, 0; V, 1+; Cr, 2+) at M062x/def2tzvpp [1-3] level

Compounds/ ions	vibrational frequencies
*$[Ca(CO)_8]^{2-}$	43.30, 43.30, 44.66, 44.66, 44.96, 44.96, 44.96, 51.73, 51.73, 51.73, 58.69, 58.69, 58.69, 197.30, 197.30, 197.30, 207.18, 211.82, 279.90, 279.90, 279.90, 296.30, 296.30, 296.30, 320.23, 320.23, 347.30, 347.30, 347.30, 376.79, 376.79, 376.79, 386.56, 386.56, 386.56, 396.47, 396.47, 1939.24, 1939.24, 1939.24, 1943.70, 1943.70, 1943.70, 1952.20, 2078.08
$[Sc(CO)_8]^-$	45.03, 45.03, 67.31, 67.31, 67.31, 69.32, 69.32, 72.31, 72.31, 72.31, 73.58, 73.58, 73.58, 226.94, 226.94, 226.94, 244.64, 262.06, 301.11, 301.11, 301.11, 310.73, 310.73, 310.73, 430.24, 430.24, 451.69, 451.69, 451.69, 455.74, 455.74, 455.74, 459.44, 459.44, 468.02, 468.02, 468.02, 2017.66, 2017.66, 2017.66, 2037.30, 2037.30, 2037.30, 2046.58, 2154.28
$[Sc(CO)_7]^-$	20.42, 20.48, 51.29, 51.87, 51.91, 58.32, 61.88, 62.09, 66.11, 70.59, 70.68, 253.14, 253.20, 270.23, 288.77, 291.56, 291.58, 324.39, 340.86, 341.74, 341.90, 372.53, 372.60, 414.25, 424.09, 424.10, 431.33, 461.89, 461.91, 485.59, 493.41, 493.43, 1997.61, 1997.61, 2004.84, 2025.94, 2032.12, 2032.12, 2143.29
$Ti(CO)_8$	45.72, 45.72, 78.65, 78.65, 78.65, 78.09, 78.09, 78.09, 84.15, 84.15, 84.17, 84.17, 84.17, 207.31, 207.31, 207.31, 219.90, 234.97, 234.97, 234.97, 269.58, 286.69, 286.69, 286.69, 471.45, 471.45, 471.45, 477.18, 477.18, 500.36, 500.36, 515.10, 515.10, 516.24, 516.24, 516.24, 2123.43, 2123.43, 2123.43, 2144.97, 2144.97, 2144.97, 2149.43, 2231.66
$Ti(CO)_7$	26.60, 26.76, 69.62, 73.01, 75.41, 75.45, 78.20, 78.21, 85.82, 89.80, 260.47, 260.51, 285.62, 307.61, 310.91, 310.93, 329.35, 351.33, 351.43, 362.05, 379.31, 379.33, 439.67, 470.02, 470.04, 492.04, 526.23, 526.27, 551.04, 561.04, 561.05, 2102.54, 2108.24, 2108.25, 2141.72, 2164.11, 2164.12, 2235.50
$[V(CO)_8]^+$	43.90, 43.90, 74.26, 74.26, 74.26,77.23, 77.23, 77.23, 83.12, 83.12, 83.12, 85.26, 85.26, 115.01, 115.01, 115.01, 135.21, 145.83, 145.83, 145.83, 219.70, 226.80, 226.80, 226.80, 396.68, 396.68, 437.21, 437.21, 437.21, 477.66, 477.66, 477.66, 497.94, 497.94, 505.03, 505.03, 505.03, 2252.88, 2252.88, 2252.88, 2262.96, 2263.33, 2263.33, 2263.33, 2307.85
$[V(CO)_7]^+$	17.48, 17.95, 75.61, 76.76, 80.08, 80.20, 89.57, 89.76, 99.63, 100.59, 100.65, 235.56, 235.58, 242.35, 278.16, 283.71, 283.78, 293.70, 308.53, 308.62, 325.69, 338.76, 339.00, 422.20, 460.95, 461.02, 493.86, 532.18, 545.68, 562.01, 562.06, 2223.03, 2233.09, 2259.53, 2287.31, 2287.31, 2316.88
$[Cr(CO)_8]^{2+}$	21.03, 37.11, 37.11, 54.83, 54.83, 54.83, 76.24, 76.24, 76.24, 79.18, 79.18, 83.61, 83.61, 83.61, 109.53, 109.53, 109.53, 125.06, 125.06, 125.06, 181.95, 201.80, 201.81, 201.81, 260.40, 260.40, 369.71, 369.71, 369.71, 397.35, 397.35, 397.35, 437.55, 437.55, 437.55, 443.87, 443.87, 2356.57, 2356.66, 2356.66
$[Cr(CO)_7]^{2+}$	4.29, 5.74, 72.34, 73.60, 74.49, 74.55, 88.63, 88.71, 97.92, 98.25, 98.29, 185.60, 201.62, 201.67, 207.78, 242.99, 243.06, 244.93, 251.08, 252.19, 263.52, 271.80, 272.08, 371.36, 402.77, 402.95, 428.15, 451.68, 451.69, 490.79, 490.87, 2360.00, 2364.34, 2364.34, 2369.76, 2381.22, 2381.22, 2387.26

** we do not find the stable conformation of $[Ca(CO)_7]^{2-}$*

We performed the calculations under the level of M062x/def2tzvpp in Gaussian 09 (E. 01 version) [1-3]

[1] Department of Chemistry, Anhui University, Hefei, Anhui 230601, P. R. China. [2] AnHui Province Key Laboratory of Chemistry for Inorganic/Organic Hybrid Functionalized Materials, Hefei, Anhui 230601, P. R. China. *email: wangkun@ahu.edu.cn; clj@ustc.edu

Supplementary Table 2. The CO dissociation energies of $[M(CO)_8]^q$ (M, q= Sc, 1-; Ti, 0; V, 1+; Cr, 2+)* at M062x/def2tzvpp level.

$M(CO)_8 \rightarrow M(CO)_7 + CO$	$\Delta_r G_m^{\ominus}$ (kcal/mol)
$[Sc(CO)_8]^- \rightarrow [Sc(CO)_7]^- + CO$	-2.65
$Ti(CO)_8 \rightarrow Ti(CO)_7 + CO$	-15.91
$[V(CO)_8]^+ \rightarrow [V(CO)_7]^+ + CO$	-21.80
$[Cr(CO)_8]^{2+} \rightarrow [Cr(CO)_7]^{2+} + CO$	-17.76

** we do not find the stable conformation of $[Ca(CO)_7]^{2-}$*

Supplementary Table 3. The comparison of Wiberg Bond Index (WBI) of metal-carbon bond analysis in $[M(CO)_8]^q$ and $[M(CO)_7]^q$ (M, q=Ca, 2-; Sc, 1-; Ti, 0; V, 1+; Cr, 2+)

M^q	Ca^{2-}**	Sc^-	Ti	V^+	Cr^{2+}
$M^q(CO)_8$	0.13	0.86	0.88	0.77	0.66
$M^q(CO)_7$	-	0.97	1.03	0.93	0.81
Ratio	-	0.886/1	0.854/1	0.828/1	0.814/1

*The ratio of the σ-bonding orbital in all the eight orbitals of $Ti(CO)_8$

** We do not find the stable conformation of $[Ca(CO)_7]^{2-}$

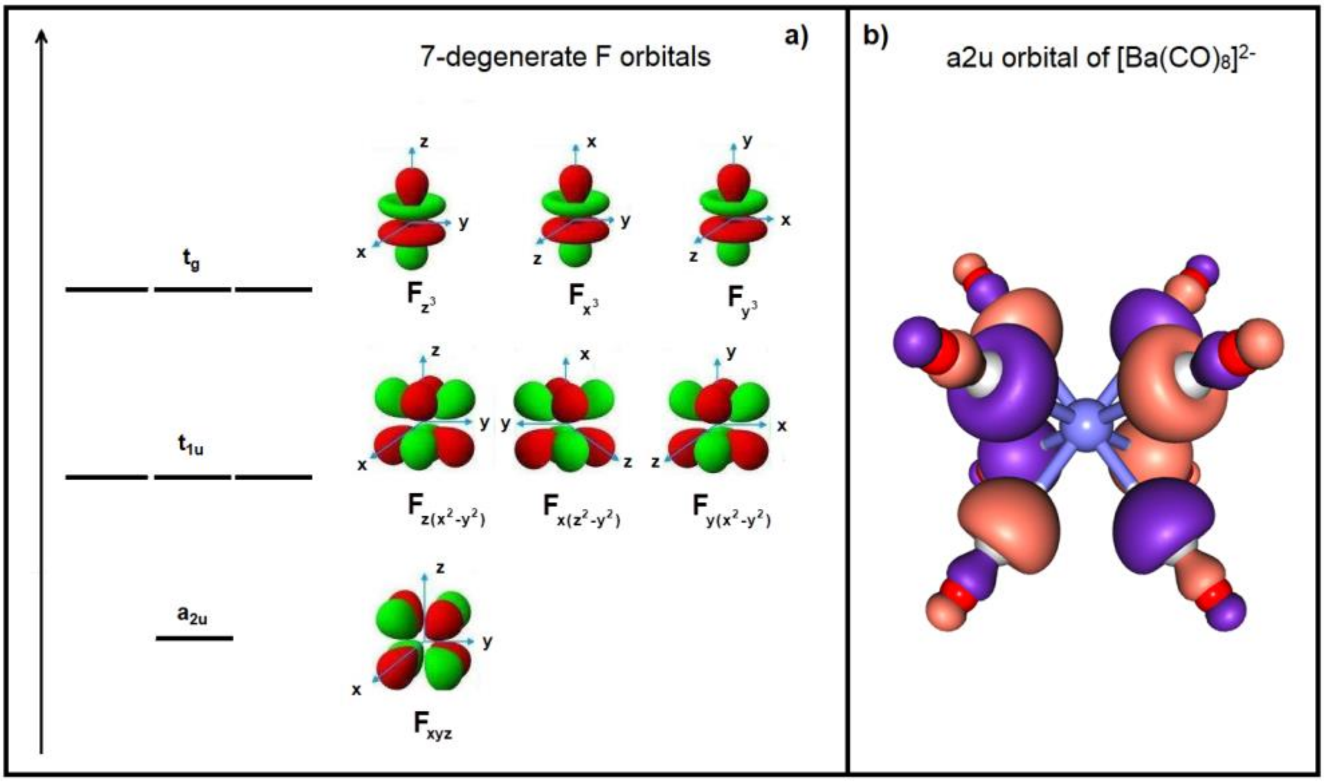

Supplementary Figure 1. The schematic diagram of 7-degenerate F orbitals. a The schematic diagram of 7-degenerate F orbitals. **b** the a2u orbital of $[Ba(CO)_8]^{2-}$

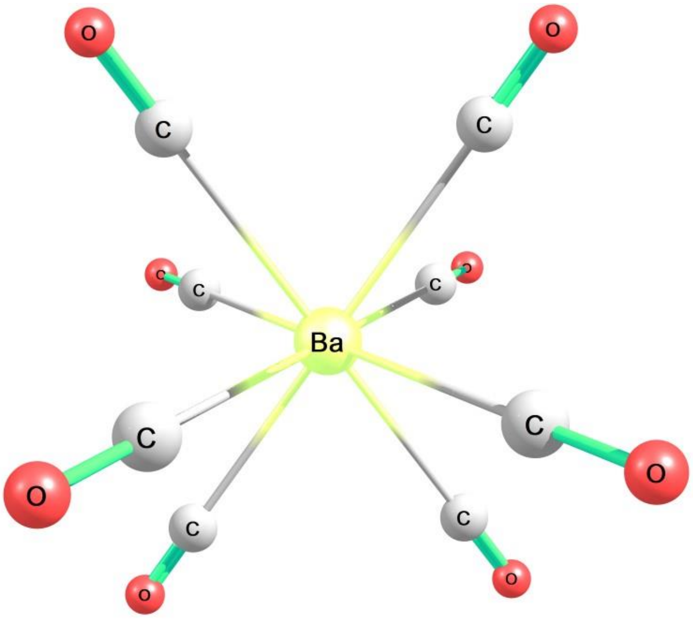

Supplementary Figure 2. The optimized structure of $[Ba(CO)_8]^{2-}$ under M06-2X/def2tzvpp theoretical level

Supplementary Table 4. The coordination of $[Ba(CO)_8]^{2-}$ under M06-2X/def2tzvpp theoretical level

Atom	x	y	z	Atom	x	y	z
C	1.681199	1.681199	1.681199	O	-2.3424	-2.3424	-2.3424
C	1.681199	-1.6812	1.681199	O	2.342397	-2.3424	-2.3424
C	1.681199	1.681199	-1.6812	O	2.342397	-2.3424	2.342397
C	-1.6812	-1.6812	-1.6812	O	2.342397	2.342397	2.342397
C	-1.6812	1.681199	-1.6812	O	-2.3424	2.342397	2.342397
C	-1.6812	1.681199	1.681199	O	-2.3424	-2.3424	2.342397
C	1.681199	-1.6812	-1.6812	O	-2.3424	2.342397	-2.3424
C	-1.6812	-1.6812	1.681199	O	2.342397	2.342397	-2.3424
Ba	0	0	0				

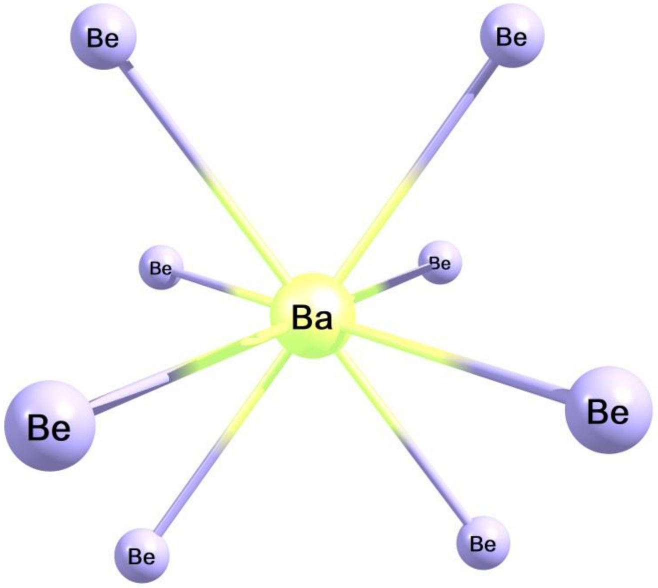

Supplementary Figure 3. The optimized structure of $[BaBe_8]^{2-}$ under M06-2X/def2tzvpp theoretical level

Supplementary Table 5. The coordination of $[BaBe_8]^{2-}$ under M06-2X/def2tzvpp theoretical level

Atom	x	y	z
Ba	0	0	0
Be	1.689905	1.689905	1.689905
Be	1.689905	-1.68991	1.689905
Be	1.689905	1.689905	-1.68991
Be	1.689905	-1.68991	-1.68991
Be	-1.68991	-1.68991	1.689905
Be	-1.68991	1.689905	1.689905
Be	-1.68991	-1.68991	-1.68991
Be	-1.68991	1.689905	-1.68991

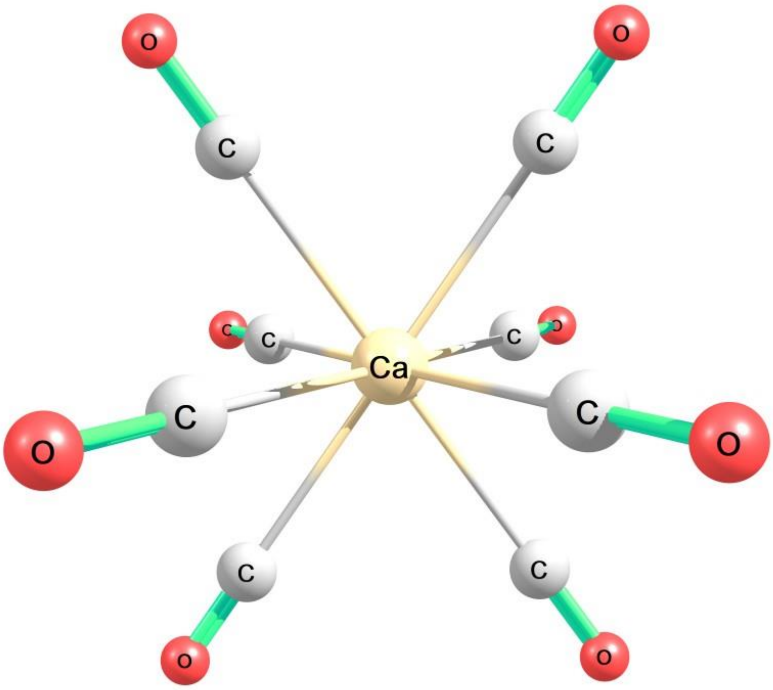

Supplementary Figure 4. The optimized structure of $[Ca(CO)_8]^{2-}$ under M06-2X/def2tzvpp theoretical level

Supplementary Table 6. The coordination of $[Ca(CO)_8]^{2-}$ under M06-2X/def2tzvpp theoretical level

Atom	x	y	z	Atom	x	y	z
C	1.455602	1.455602	1.455602	O	-2.12075	-2.12075	-2.12075
C	1.455602	-1.4556	1.455602	O	2.120749	-2.12075	-2.12075
C	1.455602	1.455602	-1.4556	O	2.120749	-2.12075	2.120749
C	-1.4556	-1.4556	-1.4556	O	2.120749	2.120749	2.120749
C	-1.4556	1.455602	-1.4556	O	-2.12075	2.120749	2.120749
C	-1.4556	1.455602	1.455602	O	-2.12075	-2.12075	2.120749
C	1.455602	-1.4556	-1.4556	O	-2.12075	2.120749	-2.12075
C	-1.4556	-1.4556	1.455602	O	2.120749	2.120749	-2.12075
Ca	0	0	0				

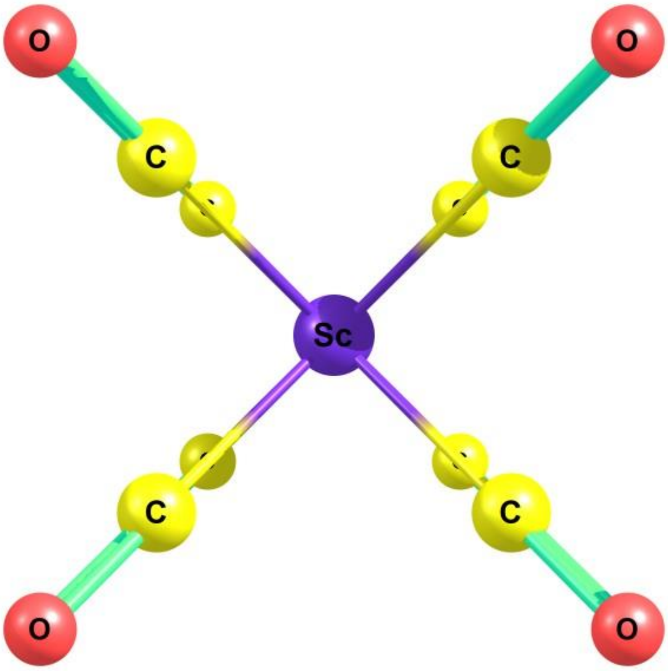

Supplementary Figure 5.The optimized structure of $[Sc(CO)_8]^-$ under M06-2X/def2tzvpp theoretical level

Supplementary Table 7. The coordination of $[Sc(CO)_8]^-$ under M06-2X/def2tzvpp theoretical level

Atom	x	y	z	Atom	x	y	z
C	1.341900	1.341900	1.341900	O	-2.001397	-2.001397	-2.001397
C	1.341900	-1.341900	1.341900	O	2.001397	-2.001397	-2.001397
C	1.341900	1.341900	-1.341900	O	2.001397	-2.001397	2.001397
C	-1.341900	-1.341900	-1.341900	O	2.001397	2.001397	2.001397
C	-1.341900	1.341900	-1.341900	O	-2.001397	2.001397	2.001397
C	-1.341900	1.341900	1.341900	O	-2.001397	-2.001397	2.001397
C	1.341900	-1.341900	-1.341900	O	-2.001397	2.001397	-2.001397
C	-1.341900	-1.341900	1.341900	O	2.001397	2.001397	-2.001397
Sc	0	0	0				

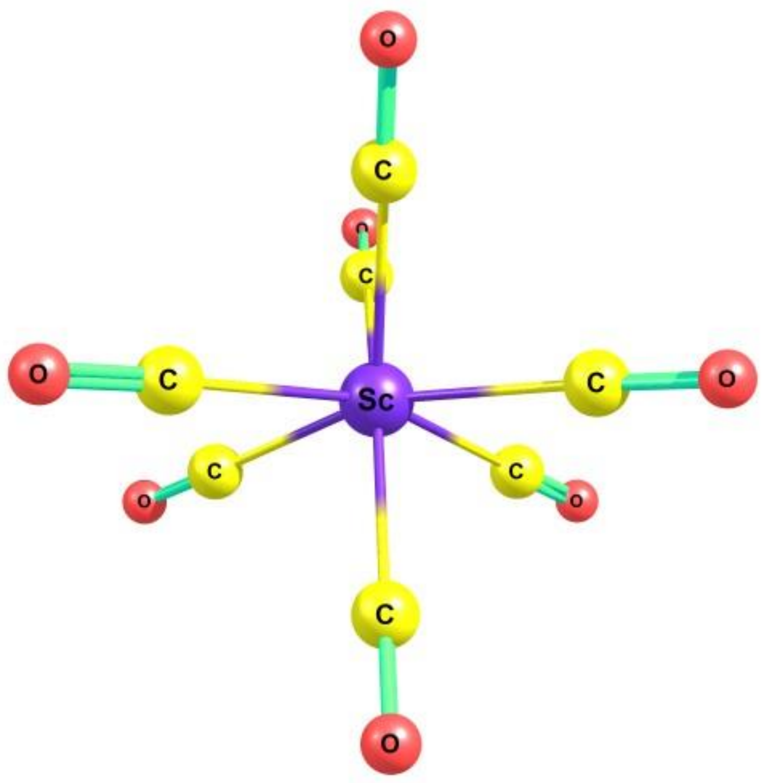

Supplementary Figure 6. The optimized structure of $[Sc(CO)_7]^-$ (C_{3v}) under M06-2X/def2tzvpp theoretical level

Supplementary Table 8. The coordination of $[Sc(CO)_7]^-$ under M06-2X/def2tzvpp theoretical level

Atom	x	y	z	Atom	x	y	z
C	-1.650997	0.953204	1.290469	O	2.844961	-1.642539	-0.827322
C	0.000000	2.169853	-0.562506	O	2.486209	1.435414	1.901834
C	1.879147	-1.084926	-0.562506	O	-2.486209	1.435414	1.901834
C	0.000000	0.000000	-2.195357	O	-2.844961	-1.642539	-0.827322
C	-1.879147	-1.084926	-0.562506	O	0.000000	-2.870827	1.901834
C	1.650997	0.953204	1.290469	O	0.000000	0.000000	-3.341760
C	0.000000	-1.906407	1.290469	O	0.000000	3.285078	-0.827322
Sc	0.000000	0.000000	0.048314				

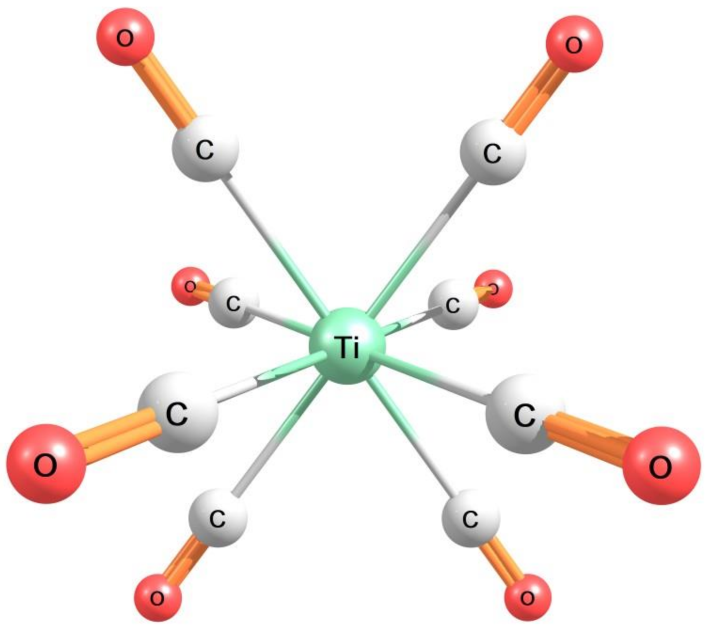

Supplementary Figure 7. The optimized structure of $Ti(CO)_8$ under M06-2X/def2tzvpp theoretical level

Supplementary Table 9. The coordination of $Ti(CO)_8$ under M06-2X/def2tzvpp theoretical level

Atom	x	y	z	Atom	x	y	z
C	1.28792	1.28792	1.28792	O	-1.94078	-1.94078	-1.94078
C	1.28792	-1.28792	1.28792	O	1.94078	-1.94078	-1.94078
C	1.28792	1.28792	-1.28792	O	1.94078	-1.94078	1.94078
C	-1.28792	-1.28792	-1.28792	O	1.94078	1.94078	1.94078
C	-1.28792	1.28792	-1.28792	O	-1.94078	1.94078	1.94078
C	-1.28792	1.28792	1.28792	O	-1.94078	-1.94078	1.94078
C	1.28792	-1.28792	-1.28792	O	-1.94078	1.94078	-1.94078
C	-1.28792	-1.28792	1.28792	O	1.94078	1.94078	-1.94078
Ti	0	0	0				

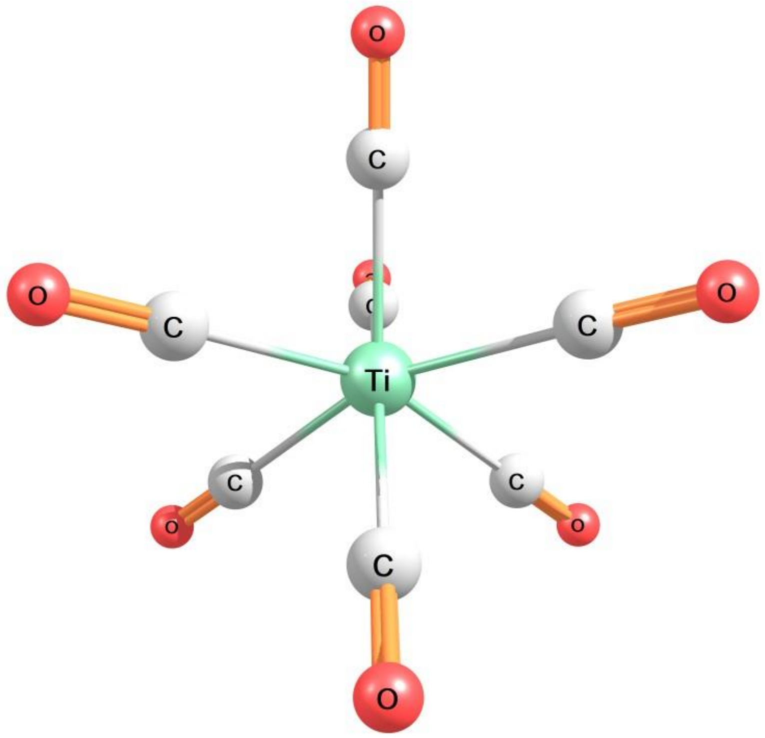

Supplementary Figure 8. The optimized structure of $Ti(CO)_7$ (C_{3v}) under M06-2X/def2tzvpp theoretical level

Supplementary Table 10. The coordination of $Ti(CO)_7$ under M06-2X/def2tzvpp theoretical level

Atom	x	y	z	Atom	x	y	z
C	-1.535221	0.886360	1.273031	O	2.338388	1.350069	1.915599
C	0.000000	2.037302	-0.577264	O	-2.338388	1.350069	1.915599
C	1.764355	-1.018651	-0.577264	O	-2.712743	-1.566203	-0.871013
C	0.000000	0.000000	-2.075523	O	-0.000000	-2.700138	1.915599
C	-1.764355	-1.018651	-0.577264	O	0.000000	0.000000	-3.211262
C	1.535221	0.886360	1.273031	O	0.000000	3.132406	-0.871013
C	-0.000000	-1.772720	1.273031	O	2.712743	-1.566203	-0.871013
Ti	0.000000	0.000000	0.024971				

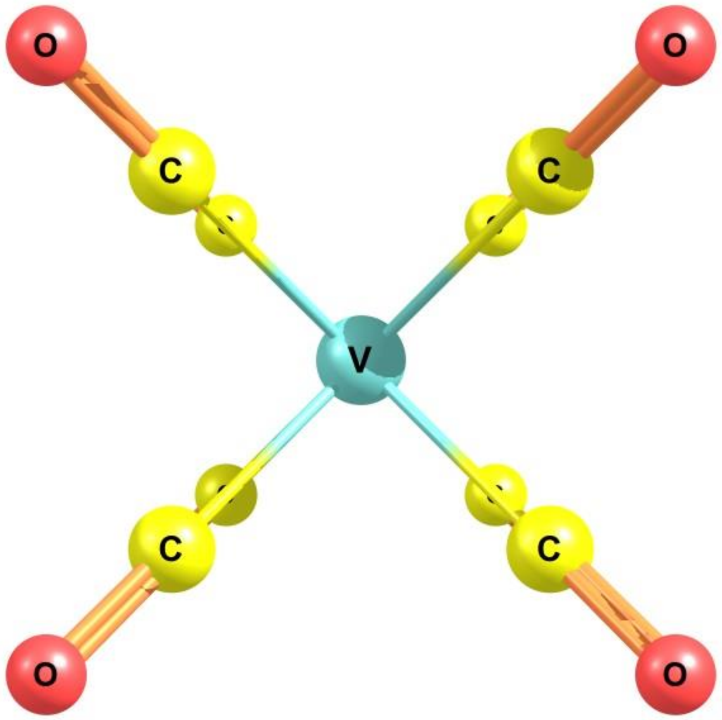

Supplementary Figure 9 The optimized structure of $[V(CO)_8]^+$ under M06-2X/def2tzvpp theoretical level

Supplementary Table 11. The coordination of $[V(CO)_8]^+$ under M06-2X/def2tzvpp theoretical level

Atom	x	y	z	Atom	x	y	z
C	1.298515	1.298515	1.298515	O	-1.944500	-1.944500	-1.944500
C	1.298515	-1.298515	1.298515	O	1.944500	-1.944500	-1.944500
C	1.298515	1.298515	-1.298515	O	1.944500	-1.944500	1.944500
C	-1.298515	-1.298515	-1.298515	O	1.944500	1.944500	1.944500
C	-1.298515	1.298515	-1.298515	O	-1.944500	1.944500	1.944500
C	-1.298515	1.298515	1.298515	O	-1.944500	-1.944500	1.944500
C	1.298515	-1.298515	-1.298515	O	-1.944500	1.944500	-1.944500
C	-1.298515	-1.298515	1.298515	O	1.944500	1.944500	-1.944500
V	0	0	0				

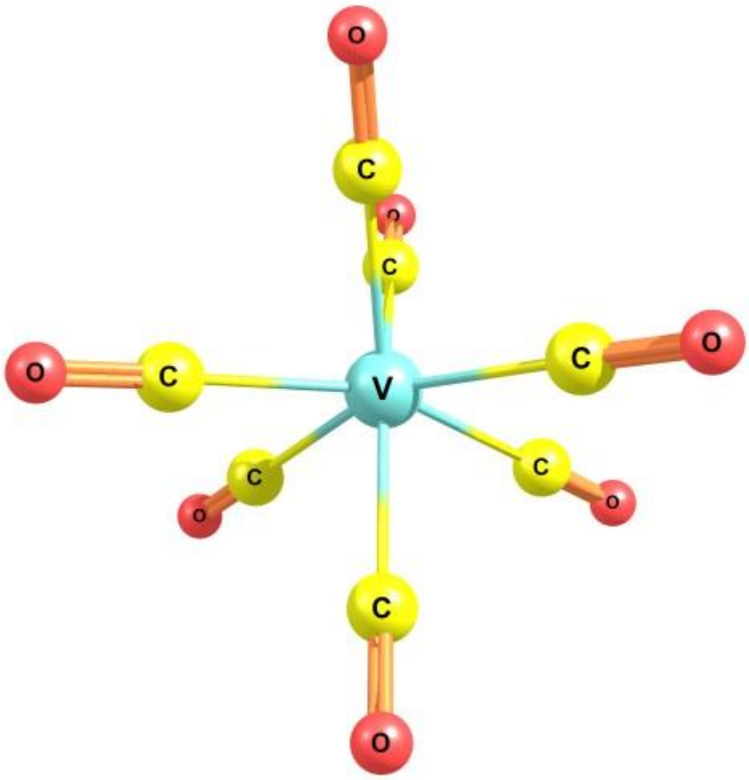

Supplementary Figure 10. The optimized structure of $[V(CO)_7]^+$ (C_{3v}) under M06-2X/def2tzvpp theoretical level

Supplementary Table 12. The coordination of $[V(CO)_7]^+$ under M06-2X/def2tzvpp theoretical level

Atom	x	y	z	Atom	x	y	z
C	-1.492840	0.861892	1.284940	O	2.668848	-1.540860	-0.892127
C	0.000000	2.000356	-0.597399	O	2.266096	1.308331	1.955180
C	1.732359	-1.000178	-0.597399	O	-2.266096	1.308331	1.955180
C	0.000000	0.000000	-2.059874	O	-2.668848	-1.540860	-0.892127
C	-1.732359	-1.000178	-0.597399	O	0.000000	-2.616663	1.955180
C	1.492840	0.861892	1.284940	O	0.000000	0.000000	-3.182561
C	0.000000	-1.723784	1.284940	O	0.000000	3.081720	-0.892127
V	0.000000	0.000000	-0.003012				

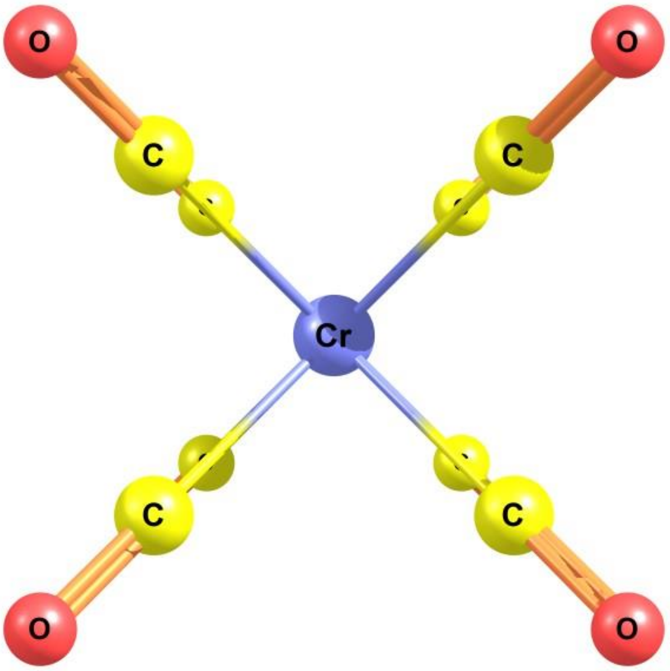

Supplementary Figure 11. The optimized structure of $[Cr(CO)_8]^{2+}$ under M06-2X/def2tzvpp theoretical level

Supplementary Table 13. The coordination of $[Cr(CO)_8]^{2+}$ under M06-2X/def2tzvpp theoretical level

Atom	x	y	z	Atom	x	y	z
C	1.35433	1.35433	1.35433	O	-1.99531	-1.99531	-1.99531
C	1.35433	-1.35433	1.35433	O	1.99531	-1.99531	-1.99531
C	1.35433	1.35433	-1.35433	O	1.99531	-1.99531	1.99531
C	-1.35433	-1.35433	-1.35433	O	1.99531	1.99531	1.99531
C	-1.35433	1.35433	-1.35433	O	-1.99531	1.99531	1.99531
C	-1.35433	1.35433	1.35433	O	-1.99531	-1.99531	1.99531
C	1.35433	-1.35433	-1.35433	O	-1.99531	1.99531	-1.99531
C	-1.35433	-1.35433	1.35433	O	1.99531	1.99531	-1.99531
Cr	0	0	0				

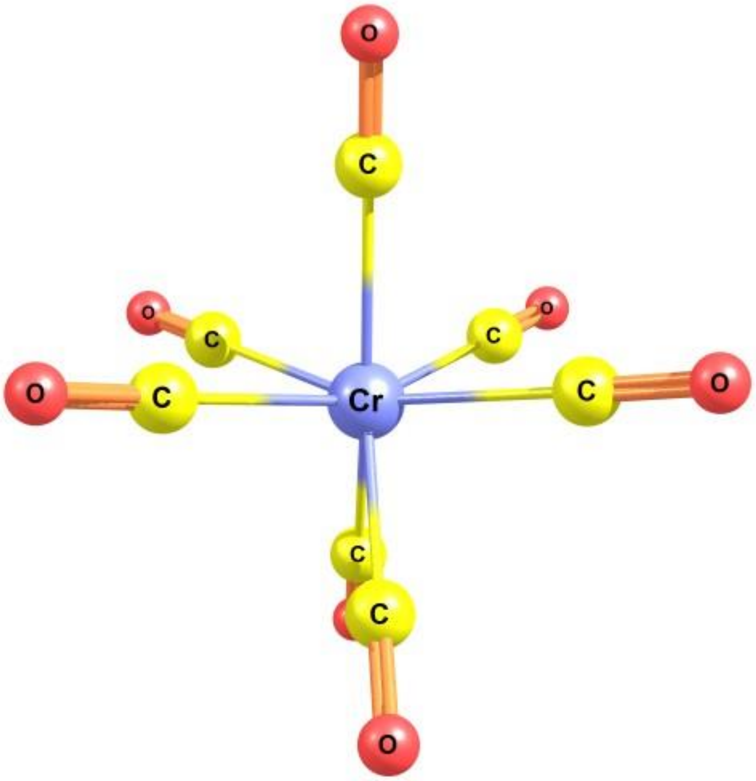

Supplementary Figure 12. The optimized structure of $[Cr(CO)_7]^{2+}$ (C_{3v}) under M06-2X/def2tzvpp theoretical level

Supplementary Table 14. The coordination of $[Cr(CO)_7]^{2+}$ under M06-2X/def2tzvpp theoretical level

Atom	x	y	z	Atom	x	y	z
C	-1.511697	0.872778	1.345239	O	2.730328	-1.576356	-0.914587
C	0.000000	2.081383	-0.626561	O	2.262023	1.305980	2.036335
C	1.802530	-1.040691	-0.626561	O	-2.262023	1.305980	2.036335
C	0.000000	0.000000	-2.176017	O	-2.730328	-1.576356	-0.914587
C	-1.802530	-1.040691	-0.626561	O	0.000000	-2.611959	2.036335
C	1.511697	0.872778	1.345239	O	0.000000	0.000000	-3.286021
C	0.000000	-1.745557	1.345239	O	0.000000	3.152711	-0.914587
Cr	0.000000	0.000000	-0.021411				

Supplementary References

1 Weigend, F. & Ahlrichs, R. Balanced basis sets of split valence, triple zeta valence and quadruple zeta valence quality for H to Rn: Design and assessment of accuracy. *Phys. Chem. Chem. Phys.* **7**, 3297-3305 (2005).

2 Zhao, Y. & Truhlar, D. G. The M06 suite of density functionals for main group thermochemistry, thermochemical kinetics, noncovalent interactions, excited states, and transition elements: two new functionals and systematic testing of four M06-class functionals and 12 other functionals. *Theor. Chem. Acc.* **120**, 215-241 (2008).

3 Frisch, M. J. T., G. W.; Schlegel, H. B.; Scuseria, G. E.; Robb, M. A.; Cheeseman, J. R.; Montgomery, J. A.; Jr.; Vreven, T.; Kudin, K. N.; Burant, J. C.; Millam, J. M.; Iyengar, S. S.; Tomasi, J.; Barone, V.; Mennucci, B.; Cossi, M.; Scalmani, G.; Rega, N.; Petersson, G. A.; Nakatsuji, H.; Hada, M.; Ehara, M.; Toyota, K.; Fukuda, R.; Hasegawa, J.; Ishida, M.; Nakajima, T.; Honda, Y.; Kitao, O.; Nakai, H.; Klene, M.; Li, X.; Knox, J. E.; Hratchian, H. P.; Cross, J. B.; Bakken, V.; Adamo, C.; Jaramillo, J.; Gomperts, R.; Stratmann, R. E.; Yazyev, O.; Austin, A. J.; Cammi, R.; Pomelli, C.; Ochterski, J. W.; Ayala, P. Y.; Morokuma, K.; Voth, G. A.; Salvador, P.; Dannenberg, J. J.; Zakrzewski, V. G.; Dapprich, S.; Daniels, A. D.; Strain, M. C.; Farkas, O.; Malick, D. K.; Rabuck, A. D.; Raghavachari, K.; Foresman, J. B.; Ortiz, J. V.; Cui, Q.; Baboul, A. G.; Clifford, S.; Cioslowski, J.; Stefanov, B. B.; Liu, G.; iashenko, A.; Piskorz, P.; Komaromi, I.; Martin, R. L.; Fox, D. J.; Keith, T.; Al-Laham, M. A.; Peng, C. Y.; Nanayakkara, A.; Challacombe, M.; Gill, P. M. W.; Johnson, B.; Chen, W.; Wong, M. W.; Gonzalez, C. & Pople, J. A. Gaussian 09. (Wallingford, 2009).
